# Successful amplification of DNA aboard the International Space Station

**DOI:** 10.1038/s41526-017-0033-9

**Published:** 2017-11-16

**Authors:** Anna-Sophia Boguraev, Holly C. Christensen, Ashley R. Bonneau, John A. Pezza, Nicole M. Nichols, Antonio J. Giraldez, Michelle M. Gray, Brandon M. Wagner, Jordan T. Aken, Kevin D. Foley, D. Scott Copeland, Sebastian Kraves, Ezequiel Alvarez Saavedra

**Affiliations:** 1 Yale University, New Haven, CT USA; 2 Department of Biology, Massachusetts Institute of Technology, Cambridge, MA USA; 3 Whitehead Institute, Cambridge, MA USA; 4 Department of Genetics, Yale University School of Medicine, New Haven, CT USA; 5 New England Biolabs, Inc., Ipswich, MA USA; 6 Boeing, Houston, TX USA; 7 miniPCR, Cambridge, MA USA

## Abstract

As the range and duration of human ventures into space increase, it becomes imperative that we understand the effects of the cosmic environment on astronaut health. Molecular technologies now widely used in research and medicine will need to become available in space to ensure appropriate care of astronauts. The polymerase chain reaction (PCR) is the gold standard for DNA analysis, yet its potential for use on-orbit remains under-explored. We describe DNA amplification aboard the International Space Station (ISS) through the use of a miniaturized miniPCR system. Target sequences in plasmid, zebrafish genomic DNA, and bisulfite-treated DNA were successfully amplified under a variety of conditions. Methylation-specific primers differentially amplified bisulfite-treated samples as would be expected under standard laboratory conditions. Our findings establish proof of concept for targeted detection of DNA sequences during spaceflight and lay a foundation for future uses ranging from environmental monitoring to on-orbit diagnostics.

## Introduction

On Earth, human health is continually improved by the use of molecular diagnostic techniques, however, these technologies remain largely untested in space conditions. It is well established that exposure to microgravity can result in profound impacts to the human body. For example, the human immune system’s function is impacted in space, and may result in a decreased response to extracellular pathogens and an altered autoimmune response.^1,2^ In order to preserve the health of astronauts during long-duration space missions, it will be important to rapidly detect molecular changes such as alterations in gene expression and epigenetic modifications. The polymerase chain reaction (PCR), a method to amplify DNA is routinely used on Earth to detect changes in DNA and gene expression and to diagnose infections among other applications.^3^ In this investigation, we sought to determine the conditions under which PCR can be carried out in microgravity to enable DNA analysis and to establish the basis for a PCR-based assay to monitor crewmember health during long-term missions. This student-led investigation resulted from the winning proposal of the first Genes in Space Competition.^4^

## Results and discussion

To test hardware performance and its capacity to amplify DNA under a variety of conditions in space, we conducted four runs in the miniPCR thermal cycler. The first ‘‘dry run’’ experiment, without biological samples, confirmed that the temperature profiles during thermal cycling were comparable to those obtained on Earth (Supplementary Fig. 1). The second and third experiments were used to determine the baseline conditions for efficient DNA amplification, and the fourth experiment attempted to detect changes in DNA methylation patterns in genomic DNA. For each experiment duplicate reactions were prepared, stored under similar conditions, and amplified on Earth in parallel with those in the International Space Station (ISS).

**Fig. 1:**
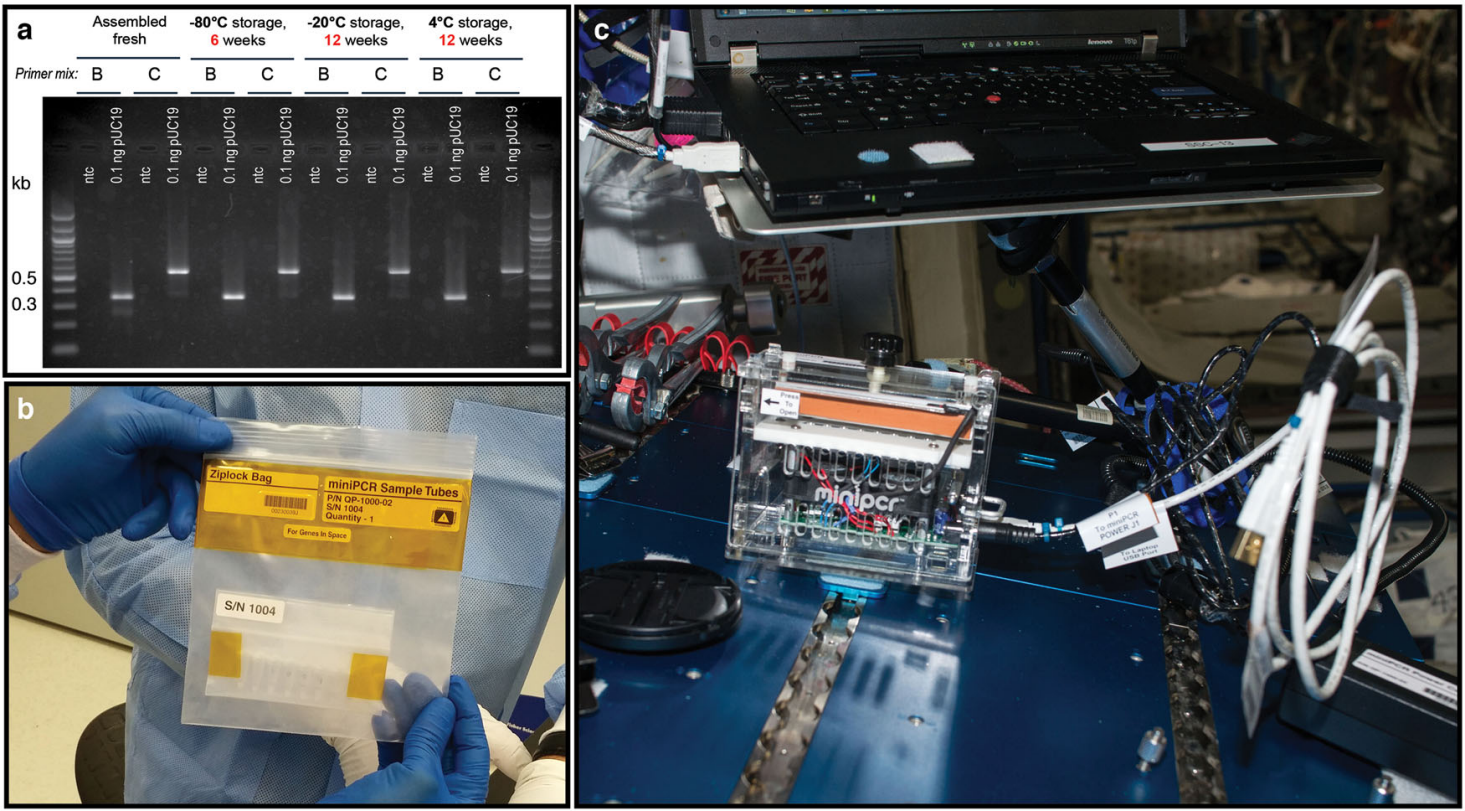
**a** Long-term stability studies. Complete reactions using Hot Start *Taq* (NEB) were prepared at the same time and stored at −80, −20, or 4 °C for either 6 or 12 weeks as indicated. Samples were then thawed or removed from the fridge, amplified using PCR and run on a 1.5% agarose gel. ntc: no template control. First and last lanes contain 0.25 μg 100 bp ladder (NEB). **b** Prepared eight-tube PCR strips were stored inside two zip bags, sealed with Kapton® tape and frozen. The samples remained frozen through all transportation steps until operations on the ISS. **c** Maintenance work area (MWA) on the ISS showing the miniPCR device connected to a laptop computer for programming and monitoring of experiments. Astronaut Tim Peake programmed and operated the miniPCR device

**Fig. 2:**
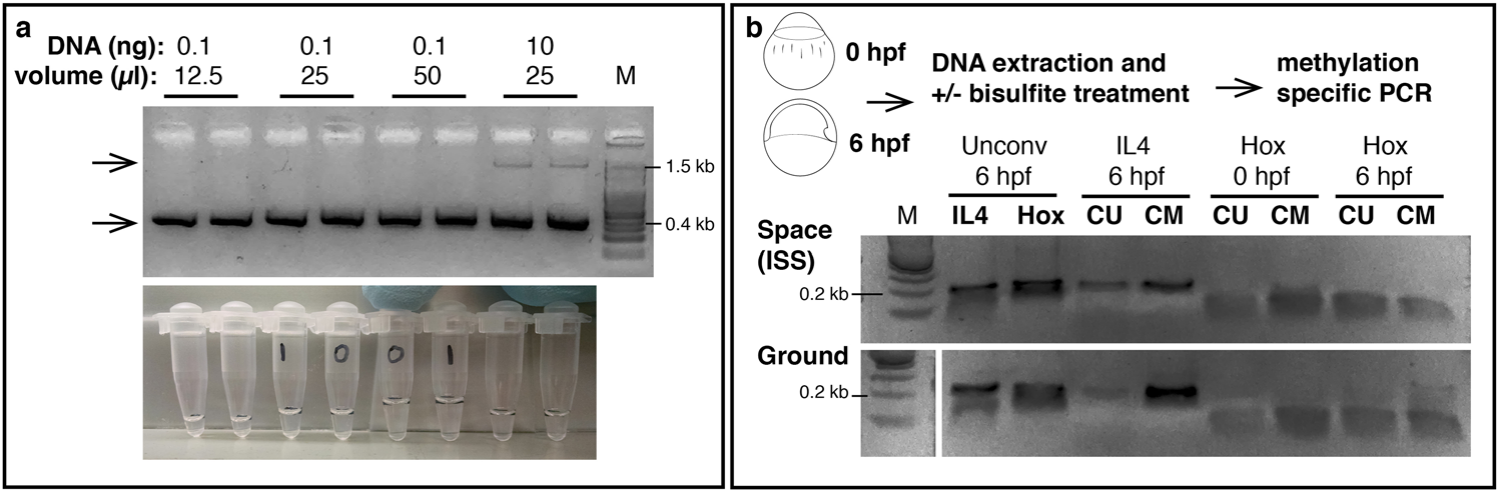
Samples were returned to Earth frozen and run on a 2% agarose gel. **a** Top: All samples with plasmid DNA were successfully amplified on ISS regardless of sample volume or amount of template DNA. Bottom arrow indicates expected amplicon size. Top arrow indicates input DNA visible in samples with 10 nanograms starting DNA. Bottom: Samples derive from the same experiment and were processed in parallel to run on the gel. Photograph of tubes corresponding to samples in the gel, showing the different reaction volumes. **b** Top: Experimental overview: *Danio rerio* embryos were harvested at 0 h and 6 h post fertilization (hpf) and genomic DNA was purified. DNA for each timepoint was divided and either left untreated or treated with sodium bisulfite. PCR was performed on untreated (unconv) DNA in lanes 2 and 3 or bisulfite-treated DNA in lanes 4 through 8. CM: primers specific to sequences that were methylated. CU: primers specific to sequences that were not methylated. DNA in lanes 2 and 3 was amplified with CU primers. Lane 1 contains 0.25 μg 100 bp ladder (NEB). Samples derive from the same experiment and were processed in parallel to run on the gel

The first experiment with a biological sample attempted to amplify plasmid DNA, as it provides a high-quality, low-complexity template that yields robust amplification on Earth. We conducted extensive stability studies of complete reactions on Earth and determined that reactions were viable after prolonged storage periods of at least 3 months (Fig. 1a), more than the time expected to lapse between sample preparation on Earth and operations at the ISS. ‘‘Complete’’ reactions, including template DNA, primers, polymerase, deoxynucleotides and reaction buffer were prepared on Earth and launched frozen on an ISS National Lab mission to the ISS^4^ (Fig. 1b). Aboard the ISS, the miniPCR device was connected to the onboard computer in the maintenance work area (MWA) for the duration of the experiment and then stowed away for later use (Fig. 1c). To determine whether optimal conditions for PCR amplification differed in the ISS environment, we varied several experimental parameters. Changes in fluid dynamics such as reduced convection, and changes in surface tension in the ISS^5,6^ might impact heat transfer throughout samples and liquid distribution in the reaction vessels, potentially impacting amplification efficiency due to incomplete DNA denaturation, poor primer annealing, or reduced polymerase efficiency. To determine whether the altered fluid dynamics would impact DNA amplification, we prepared reactions in total volumes of 12.5, 25, and 50 µl, which span the range of volumes typically used in laboratories. We also varied the amount of template DNA between 0.1 and 10 ng, and utilized two different DNA polymerases, *Taq* polymerase and Q5® polymerase. We found that DNA amplification in the ISS was successful under all experimental conditions tested (Fig. 2a), indicating that PCR can be conducted aboard the ISS without modification of standard protocols. Since the Genes in Space-1 studies were performed, two other independent efforts, NASA WetLab-2 and Water Monitoring Suite, have also amplified DNA aboard the ISS (unpublished).

It is becoming increasingly clear that altered epigenetic profiles can lead to a variety of disorders, and that spaceflight produces measurable changes to the epigenome.^7,8^ An assay to evaluate epigenetic changes such as DNA methylation during spaceflight would aid our understanding of the effects of spaceflight on the epigenome and would allow monitoring of crewmembers’ health. For example, such an assay could aid in the detection of immune system alterations that may to lead to increased susceptibility to autoimmune disease, allergies, and other diseases in space.^9,10^ The standard method used to evaluate the methylation profile of DNA is bisulfite conversion.^11^ During bisulfite treatment unmethylated cytosines are converted to uracil through deamination, while methylated cytosines are protected from the conversion. These resulting alterations can be revealed using primers specific to converted or non-converted sequences.^12^ We sought to determine whether such epigenetic changes could be detected using PCR technology on the ISS through the use of methylation-specific primers. We used zebrafish embryos as an experimental system because its methylation patterns have been studied in detail at a genome-wide level.^13^ We selected to study the promoters of *Hoxb3a*, a developmental gene, and *Interleukin 4* (*IL4*), an immune gene, as the methylation patterns for these genes have been shown to change in embryos between 0 and 6 h post-fertilization. DNA for each time point was prepared, divided and either left untreated or treated with sodium bisulfite. Complete amplification reactions to detect converted and non-converted DNA were prepared on Earth and sent frozen to ISS. PCR performed on untreated DNA (Fig. 2b, lanes 2 and 3) resulted in robust amplification of *IL4* and *Hox* in space samples and ground controls. At 6 hpf *IL4* space-amplified samples with primers specific to sequences that were methylated (Fig. 2b, top gel, lane 5) amplified more robustly than samples with primers specific to sequences that were not methylated (Fig. 2b, top gel, lane 4). These results mirror the ones from samples amplified on Earth (Fig. 2b, bottom gel, lanes 4 and 5), suggesting that methylation-specific PCR can be conducted in space without modifications. Reactions targeting the Hox promoter that contained bisulfite-treated DNA resulted in poor amplification in both space samples and ground controls (Fig. 2b, lanes 6–9), likely due to the increased fragility of bisulfite-treated DNA.^14^

Taken together, our experiments reveal that PCR can be robustly carried out in a microgravity environment without adaptations to the hardware, consumables, or reagents, opening the door to DNA analysis in space. Additionally, we found that differences in DNA methylation are detectable in this microgravity environment. Furthering our ability to evaluate molecular changes in space is essential to both our understanding of the effects of spaceflight on the human body and the maintenance of astronaut health to enable long-term missions. Recent reports of DNA sequencing aboard the ISS using samples prepared on Earth,^15^ in combination with the successful on-orbit DNA amplification reported in this article, suggest that a complete sample-to-sequence DNA analysis workflow in space will be plausible in the near future.

## Materials and methods

### Equipment and consumables used for PCR

The mini8 miniPCR is manufactured by miniPCR (www.minipcr.com). The 0.2 ml PCR tubes were purchased from Eppendorf (Cat. No. 0030124359) and consist of 8-tube strips with hinged lids.

### Long-term stability studies

‘‘Complete’’ reactions containing 25 µl Q5® Hot Start High-Fidelity 2X Master Mix (NEB M0494), 5 µl primer mix (5 μM each), 19 µl water and 1 µl 0.1 ng/μl pUC19 (see below) were stored at the indicated temperatures for 6 or 12 weeks. pUC19 was replaced for water in no template controls. Samples were then thawed and immediately amplified. Two sets of primers, B and C, were tested (primer sequences in Supplementary Methods). Primer set B was selected for experiments conducted in ISS and ground controls. pUC19 was obtained in purified form from New England Biolabs (N3041). pUC19 is isolated from *E. coli* ER2272 (dam^+^ dcm^+^ EcoK M^−^) by a standard plasmid purification procedure. Amplification conditions for long-term stability studies were as following: 98 °C/30 s [98 °C/15 s, 64 °C/15 s, 72 °C/60 s]x30, 72 °C/5 min.

### Space plasmid amplification

For space samples, complete reactions were prepared as described above and placed in 0.2 ml PCR tubes in 12.5, 25, and 50 µl aliquots as indicated. Samples were kept frozen until operations aboard the ISS. Amplification conditions for space samples and ground controls were as following: 95 °C/30 s [95 °C/15 s, 50 °C/15 s, 68 °C/60 s]x30, 68 °C/5 min.

### DNA purification from Danio rerio embryos

Zebrafish lines were maintained in accordance with AAALAC research guidelines, under a protocol approved by Yale University IACUC. Embryos were obtained from natural crosses of TU-AB and TLF strains, with mating pairs selected from random pools of wild-type females and males.

DNA was extracted from embryos using previously described nuclei isolation protocols.^13^ In brief, embryos were first chemically dechorionated (Pronase 1 mg/ml) and allowed to develop till the desired stage. Batches of embryos were disrupted in 1 ml lysis buffer (10 mM Tris-Cl [pH 8.0], 10 mM NaCl, 0.5% NP-40) using a 20 G needle. Nuclei were collected by centrifugation at 3500×*g* for 5 min at 4 °C. Unless otherwise noted, 500 and 100 embryos were used for 0 hpf and 6 hpf, respectively. Nuclei were resuspended in 1 ml nuclei lysis buffer (50 mM Tris-Cl [pH 8.0], 10 mM EDTA, 1% SDS) with 1 µl of RNase Cocktail (100 mg/ml, QIAGEN) and 10 μl Proteinase K Solution (Invitrogen). Following a 10-minute incubation at 55 °C, equal volume phenol:chloroform (Invitrogen) was added and samples centrifuged at 4 °C for 30 min at 14,000 rpm. The aqueous phase was collected and transferred to a new microcentrifuge tube. To further reduce the large maternal messenger RNA contribution, 1 µl of RNase Cocktail was added and samples incubated for at least 3 h at 37 °C. A second phenol:chloroform extraction was performed as detailed above and DNA precipitated by adding 1.2 volume of isopropanol and 1/10 volume 3 M sodium acetate at −80 °C for one hour. Samples were centrifuged at 4 °C for 30 min at 14,000 rpm and the supernatant discarded. Afterwards, the DNA pellet was twice washed in 70% ethanol and spun at 14,000 rpm for 15 min at 4 °C. The DNA pellet air dried for 10 min at room temperature and was resuspended in nuclease-free water.

### Bisulfite treatment

Sodium bisulfite treatment was performed using the EZ DNA methylation lightning reagents (Zymo) according to the manufacturers recommendations. Briefly, 500 ng of DNA was denatured and converted by incubating at 98 °C for 8 min followed by a 54 °C, 60 min step using the conversion reagent. Desulphonation and cleanup of converted DNA was performed using a DNA-binding spin column. Converted DNA was eluted in 10 µl of nuclease-free water.

### Methylation-specific PCR of Danio rerio genomic DNA

‘‘Complete’’ reactions containing 12.5 µl Q5® dU Bypass Master Mix (2x) (NEB M0598), 5 µl primer mix (5 μM each), 6.5 µl water and 1 µl genomic DNA. Samples were kept frozen until operations aboard the ISS. Amplification conditions for space samples and ground controls were as following: 94 °C/60 s [94 °C/10 s, 63 °C/30 s, 72 °C/30 s]x35, 72 °C/2 min.

### Sample preparation and ISS operations

The Genes in Space-1 samples flew to the ISS on a Space X Dragon vehicle mated to a Falcon rocket. The samples were prepared on the ground and then delivered frozen to the Kennedy Space Center (KSC) as ‘‘Late Load.’’ Samples were kept frozen during transit using −20 °C phase change cold packs (Cryopak) and were delivered to KSC approximately 3 days before launch where they were kept in the POLAR freezer on the Dragon vehicle with a set point of −35 °C. At ISS samples were placed in the MELFI freezer at −95 °C and stored until the miniPCR run would take place.

During operations, ISS crew removed the sample from the freezer and allowed 5 min for it to thaw before placing in the miniPCR. Parameters for thermal cycling were programmed by crew through the onboard computer and uploaded to the miniPCR via a USB cable. After upload the operations continued unattended. When the run was complete, the miniPCR data from the run was saved and all power was removed. After 30 min to allow for cool down, the sample was removed from the miniPCR and placed back into an ISS freezer (GLACIER), where it stayed until loaded back into a Space X Dragon for return. Samples were returned in an unpowered coldbag that was held at +4 °C via coldbricks. Upon splashdown, the vehicle was unloaded and the samples were put into a freezer for return to the Johnson Space Center, then turned over to the Boeing team and packaged in Fedex cold storage shipper (ice packs) for return to miniPCR for final analysis.

### Data availability

All data generated or analyzed during this study are included in this published article (and its supplementary information files).

## Electronic supplementary material

Supplementary Materials for Successful amplification of DNA aboard the International Space Station

Supplementary Figure 1

Supplementary Figure 2

Supplementary Figure 3

## References

[1] Mermel LA (2013). Infection prevention and control during prolonged human space travel. Clin. Infect. Dis..

[2] Sonnenfeld G, Shearer WT (2002). Immune function during space flight. Nutrition.

[3] Powledge TM (2004). The polymerase chain reaction. Adv. Physiol. Educ..

[4] Genes in Space-1. https://www.nasa.gov/mission_pages/station/research/experiments/1913.html. (Accessed: 2nd July 2017)

[5] Straub J (1994). The role of surface tension for two-phase heat and mass transfer in the absence of gravity. Exp. Therm. Fluid Sci.

[6] Napolitano LG (1984). Materials: marangoni convection in space microgravity environments. Science.

[7] Ou X (2010). Spaceflight-induced genetic and epigenetic changes in the rice (*Oryza sativa L*.) genome are independent of each other. Genome.

[8] Tauber S, Yi B, Chouk r A, Ullrich O (2016). Effect of Spaceflight and Spaceflight Analogue Culture on Human and Microbial Cells.

[9] Yi B, Crucian B, Tauber S, Ullrich O, Chouk r A (2016). Effect of Spaceflight and Spaceflight Analogue Culture on Human and Microbial Cells.

[10] Taylor, G. R., Konstantinova, I., Sonnenfeld, G. & Jennings, R. in *Adv. Space Biol. Med*. **6**, 1–32 (1997). 10.1016/s1569-2574(08)60076-3 9048132

[11] Zuo T, Tycko B, Liu TM, Lin JJL, Huang THM (2009). Methods in DNA methylation profiling. Epigenomics.

[12] Herman, J. G. Approaches, Methods, and Applications. in *DNA Methylation,* (ed. Manel Esteller) 65–72 (CRC Press, 2004). 10.1201/9780203487013.ch5.

[13] Potok ME, Nix DA, Parnell TJ, Cairns BR (2013). Reprogramming the maternal zebrafish genome after fertilization to match the paternal methylation pattern. Cell.

[14] Mill, J. & Petronis, A. Profiling DNA Methylation from Small Amounts of Genomic DNA Starting Material: Efficient Sodium Bisulfite Conversion and Subsequent Whole-Genome Amplification in *DNA Methylation Methods in Molecular Biology* (ed. Tost J, Vol. 507) 371–391 (Humana Press, 2009). 10.1007/978-1-59745-522-0_27 18987828

[15] McIntyre ABR (2016). Nanopore sequencing in microgravity. Npj Microgravity.

