# Author Correction: Successful amplification of DNA aboard the International Space Station

**DOI:** 10.1038/s41526-017-0035-7

**Published:** 2018-01-10

**Authors:** Anna-Sophia Boguraev, Holly C. Christensen, Ashley R. Bonneau, John A. Pezza, Nicole M. Nichols, Antonio J. Giraldez, Michelle M. Gray, Brandon M. Wagner, Jordan T. Aken, Kevin D. Foley, D. Scott Copeland, Sebastian Kraves, Ezequiel Alvarez Saavedra

**Affiliations:** 1 Yale University, New Haven, CT USA; 2 Department of Biology, Massachusetts Institute of Technology, Cambridge, MA USA; 3 Whitehead Institute, Cambridge, MA USA; 4 Department of Genetics, Yale University School of Medicine, New Haven, CT USA; 5 New England Biolabs, Inc., Ipswich, MA USA; 6 Boeing, Houston, TX USA; 7 miniPCR, Cambridge, MA USA

**Correction to:** *npj Microgravity* (2017); doi:10.1038/s41526-017-0033-9; Published 16 November 2017

The following text was added to the Author Contributions section: A.S.B., H.C.C., and A.R.B. contributed equally to the work and are considered co-first authors. This has now been corrected in the HTML and PDF versions of this Article.

