# Supplementary material for: Successful amplification of DNA aboard the International Space Station
Source: NPJ Microgravity. 2017 Nov 16;3:26. doi: 10.1038/s41526-017-0033-9 (PMC5691047; doi:10.1038/s41526-017-0033-9)
Supplement: Supplementary file 1 — Supplementary Materials for Successful amplification of DNA aboard the International Space Station [file 41526_2017_33_MOESM1_ESM.docx]

Supplementary Materials for

**Successful amplification of DNA aboard the International Space Station**

Anna-Sophia Boguraev^1^, Holly C. Christensen^2^, Ashley R. Bonneau^3^, John A. Pezza^4^, Nicole M. Nichols^4^, Antonio J. Giraldez^3^, Michelle M. Gray^5^, Brandon M. Wagner^5^, Jordan T. Aken^5^, Kevin D. Foley^5^, D. Scott Copeland^5^, Sebastian Kraves^6^ and Ezequiel Alvarez Saavedra^6^

Supplementary Figure Legends

Supplementary Figure 1. miniPCR 'Dry run'. Temperature profile during thermal cycling on ISS and ground controls. Run conditions for ISS and ground controls were as following: 94°C/30 sec [94°C/15 sec, 63°C/15 sec, 72°C/15 sec]x10, 72°C/5 sec.

Supplementary Figure 2. Full gel corresponding to Figure 2a

Supplementary Figure 3. Full gel corresponding to Figure 2b

Supplementary Methods

Primer sequences used in this study:

*Plasmid DNA*

Left primer B CGGCATCAGAGCAGATTGTA

Right primer B AGCGGATAACAATTTCACACAGGA

Left primer C CGCCAGGGTTTTCCCAGTCACGAC

Right primer C TTTTTGTGATGCTCGTCAGG

***hoxb3a***

Primers used on bisulfite treated DNA:

CM

Left  M primer  GTGTCGTTGTTATTTTTATTGTTTC

Right M primer AAATAAAATACTTTCGTCCAATACG
Product size: 229

CU

Left  U primer  GTTGTTGTTATTTTTATTGTTTTGT

Right U primer ATAAAATACTTTCATCCAATACACA

Primers used on untreated DNA (intron):

Left primer   TCTGACATTGTCTCGCAACC
Right primer   GGGAAACACAAGGAAAAGCA

***IL4***

Primers used on bisulfite treated DNA:

CM

Left  M primer   GTGTTTGTGGTTTTTTATTGATTC
Right M primer  ATAAAATATACCTATTTTAATCGCA
Product size: 159

CU

Left  U primer  GTTTGTGGTTTTTTATTGATTTGG

Right U primer CATATAAAATATACCTATTTTAATCACA
Product size: 160

Primers used on untreated DNA (intron):

Left primer   TGCCACACCTTCTGTAGCAC
Right primer   TGTGGATAAGCGCAATGATG
