# Supplementary figures and images for: Successful amplification of DNA aboard the International Space Station
Source: NPJ Microgravity. 2017 Nov 16;3:26. doi: 10.1038/s41526-017-0033-9 (PMC5691047; doi:10.1038/s41526-017-0033-9)

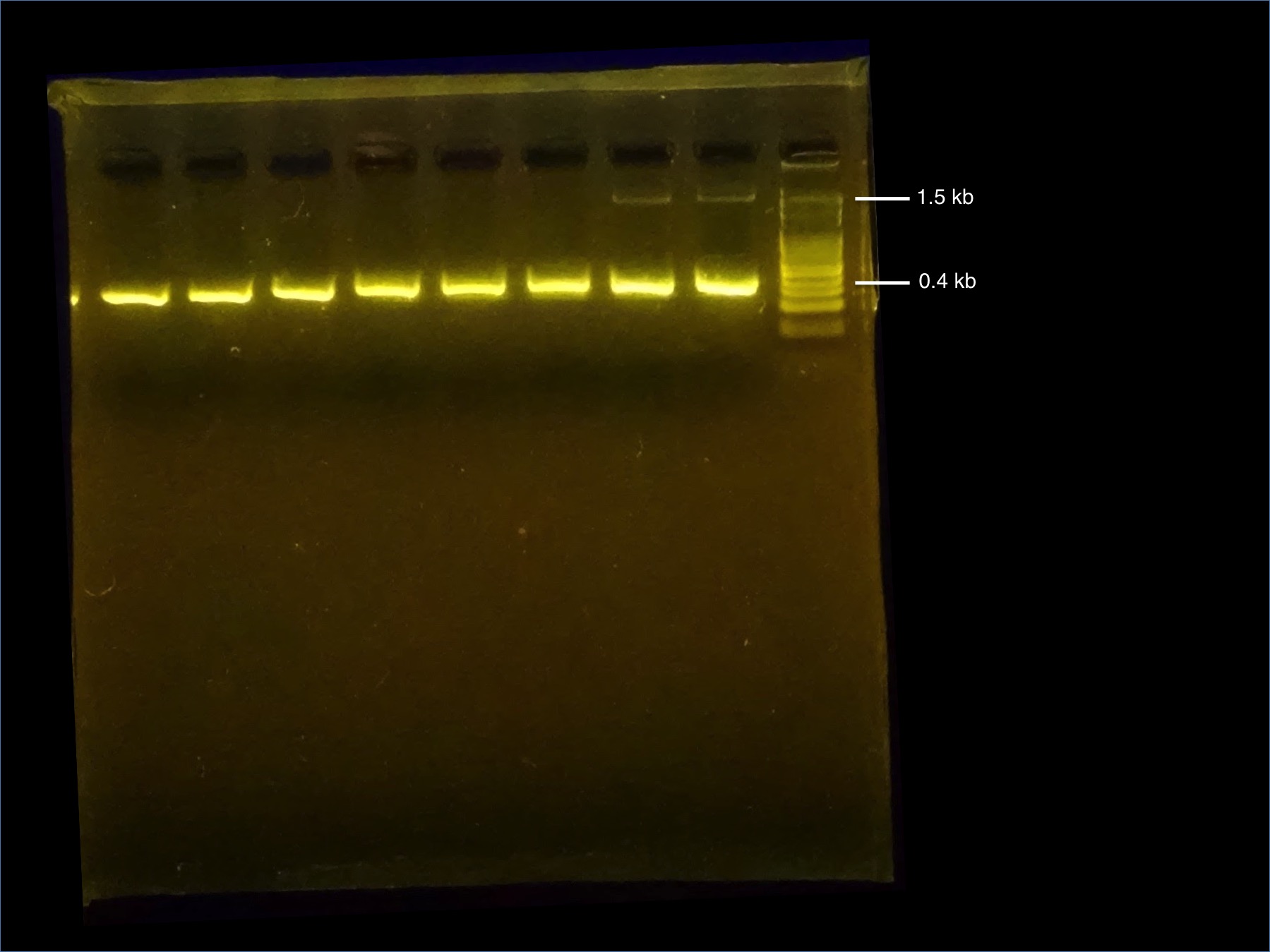

Supplement: Supplementary file 3 — Supplementary Figure 2 [file 41526_2017_33_MOESM3_ESM.jpg]

**Space  
(ISS)**

**Earth  
(Control)**

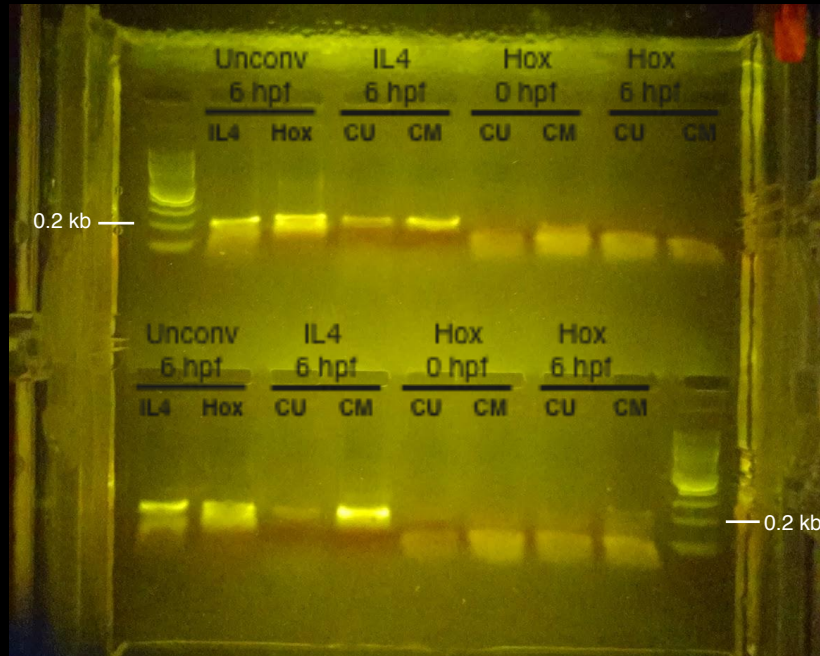

Supplement: Supplementary file 4 — Supplementary Figure 3 [file 41526_2017_33_MOESM4_ESM.pdf]
